# Distribution of Trace Metals in Ice and Water of Liaodong Bay, China

**DOI:** 10.3390/ijerph192215241

**Published:** 2022-11-18

**Authors:** Weijun Guo, Sihong Liu, Xiangpeng Kong, Lixin Sun, Jibing Zou

**Affiliations:** 1 College of Environmental Sciences and Engineering, Dalian Maritime University, Dalian 116026, China; 2 The State Key Laboratory of Coastal and Offshore Engineering, Dalian University of Technology, Dalian 116024, China

**Keywords:** trace metal, sea ice, partitioning coefficient, Liaodong Bay

## Abstract

Trace metal pollution in coastal seas has been of great concern because of its persistence, toxicity, and biological accumulation through the food chain. The role of sea ice in trace metal transport and distribution in Liaodong Bay is still unknown. Sea ice and water samples were collected in Liaodong Bay in February 2021 to assess the distributions of Cu, Pb, Cd, Zn, Cr and Hg during the frozen season. Total dissolved (<0.45 μm) and particulate (>0.45 μm) heavy metal concentrations were measured by atomic absorption spectrophotometry (Cu, Pb, Cd, Zn and Cr) and atomic fluorescence spectrophotometer (Hg). The ice held significantly higher levels of total Cr when compared to water. There were no significant differences in total concentrations of Cu, Pb, Cd, Zn and Hg between water and ice samples. An analysis of dissolved-to-total metal ratios shows that all studied metals in the dissolved phase, except Hg, are found exclusively in Liaodong Bay nearshore ice as a result of desalination. Concentrations of particulate metals are higher in sea ice than in seawater due to suspended/bed sediment entrainment and atmospheric deposition. The partitioning coefficients of six trace metals are not increased with the increase in the concentration of particulate matter in sea ice due to sediment accumulation. The redistribution of trace metals between seawater and ice was a result of comprehensive effects of physico-chemical processes and environmental factors, such as chemical oxygen demand, salinity, and suspended particulate material.

## 1. Introduction

Sea ice occurs in approximately 10% of the world ocean’s surface, and the ice-covered area varies between 8 × 10^6^ and 15 × 10^6^ km^2^ in the northern hemisphere [1]. An enormous amount of research effort on sea ice has focused on its impact on shipping safety and offshore platform risks [2,3]. With the increasing frequency of human activities in the polar region, the interaction between sea ice and the ecological environment has received increasing attention [4,5].

Sea ice has a significant impact on the biogeochemical processes in cold regions. Besides heat and salinity, the formation, drift and melting of ice contribute to the redistribution of the pollutants in marine systems. When the sea water freezes into ice, the salt will remain in the water due to the repulsive effect. Therefore, the formation of sea ice will increase the salinity of a region, while the melting of ice often has the opposite effect.

Unlike the desalination process of the rejection of salt, sea ice seems to accumulate trace metals [6]. Dissolved heavy metal levels measured in Arctic ice were extensively compared to mean concentrations in surface seawater [7]. Sediment-laden sea ice, formed as a result of ice scouring action and atmospheric deposition, often provides bioavailable iron for iron-deficient sea [8,9]. Free-drifting icebergs carrying accumulated metals are widely perceived as sources of dissolved iron for the polar seas [9,10].

Direct releases from the ice of particulate heavy metals, such as Fe and Cu, cause the surface concentration of seawater to capture a peak quickly after the pack ice melts [11]. Laboratory experiments by Janssens et al. (2018) indicate that dissolved Fe is preferentially enriched into sea ice compared to particulate Fe, and that the high organic ligands contribute to the incorporation process of dissolved Fe [12].

Although research on both the Arctic and Antarctic regions demonstrates that sea ice accumulates trace metals, the enrichment effect of heavy metals in other regions does not always work [13]. Laboratory experiments by Sun et al. (2018) indicated that more iron was distributed into the under-ice water rather than the freezing ice during the process of water icing, which can be due to the lower binding energy in water than in ice [14]. Laboratory experiments by Tang et al. (2020) indicated that iron partitioned at a greater rate into the under-ice water rather than the freezing ice during the water freezing process. The partition coefficient decreases with the increase in ice thickness, initial concentration, and freezing temperature [15]. Both the conclusions of Sun et al. (2018) and Tang et al. (2020) are based on the freshwater experiments, and the metals are of a pure ionic state. Most of the dissolved metals in the coastal environments that exist bind to strong organic ligands rather than the ionic state [16].

The present attention has primarily been focused on the polar seas, which are covered by sea ice for most of the year. In some mid-latitude sea areas, sea ice also appears every winter. The most southerly site in the northern hemisphere where an extensive sea ice cover forms is the Bohai Sea, located off the east coast of China between the latitudes of 37° and 41° N. As one of three bays of the Bohai Sea, Liaodong Bay has the longest frozen period, which normally lasts 3–4 months from November to February. During a mild winter, ice forms only in the northern part or nearshore of Liaodong Bay, but during a cold winter the whole Liaodong Bay is covered in ice. Liaodong Bay is a severely impacted urban estuary, as inputs of contaminants to the estuary from riverine sources, urban sewage discharge, agricultural runoff, maritime vessels, atmospheric deposition and accidental spills have become increasingly significant over the past five decades.

At present, there is a great deal of research on the spatial distribution, temporal evolution and water–sediment exchanging process of heavy metals in the Liaodong Bay [17,18,19]. However, there are few studies on the partition and migration processes of heavy metals between ice–water systems. The growth and decay of sea ice is the most prominent physical process of Liaodong Bay in winter, so its impact on the transport and distribution of environmental pollutants deserves detailed study.

The present work provides new data on the spatial distribution and partitioning between water and ice of six heavy metals (copper (Cu), lead (Pb), cadmium (Cd), zinc (Zn) chromium (Cr), and mercury (Hg)) in Liaodong Bay. Its specific aims are to (i) assess how ice influences behavior of different trace metals and their fractions; (ii) examine if the coastal ice is able to contribute to accumulating high levels of trace metals; (iii) analyze factors affecting trace metal distribution.

## 2. Materials and Methods

### 2.1. Study Area and Sample Collection

**Figure 1:**
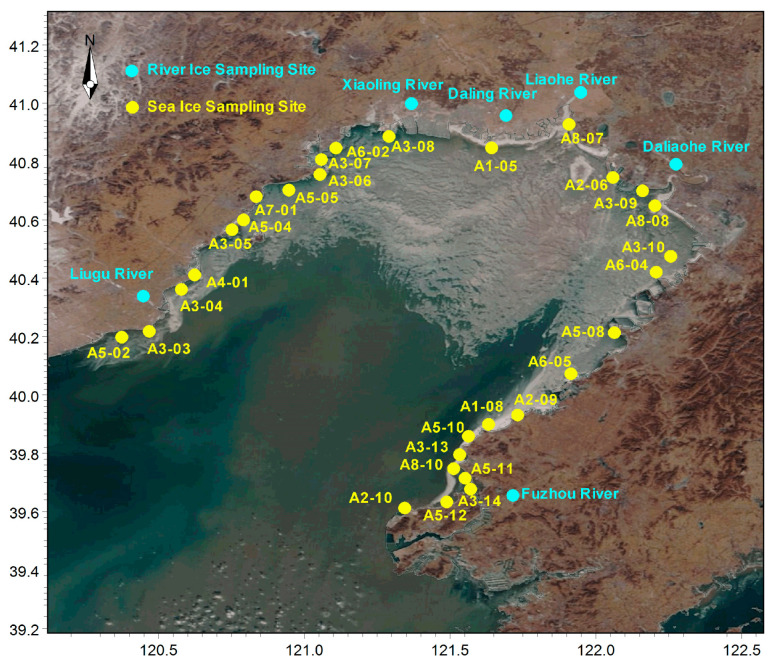
Sampling site in the coastal area of Liaodong Bay.

According to the national marine functional zoning and the marine functional zoning of Liaoning Province, the coastal waters of Liaodong Bay are selected as the survey area (Figure 1), and the survey time is February 2020 (winter). There are 30 sampling sites along the coast, distributed in 8 types of marine functional areas: aquaculture/fishing zone (A1-05, A1-08), harbor/shipping zone (A2-06, A2-09, A2-10), industry/urban sea zone (A3-03, A3-04, A3-05, A3-06, A3-07, A3-08, A3-09, A3-10, A3-13, A3-14), mineral/energy extraction zone (A4-01), tourism/entertainment zone (A5-02, A5-04, A5-05, A5-08, A5-10, A5-11, A5-12), marine protected zone (A6-02, A6-04, A6-05), reserved zone (A7-01), and specific utility zone (A8-07, A8-08, A8-10). Samples of fast ice and underflow were collected and measured in our research.

### 2.2. Analytical Method

The recovered ice samples were melted in light-proof conditions at room temperature. All sampling, on-site treatment and analysis methods were performed with reference to the Marine Monitoring Specification Part 4: Seawater Analysis and were strictly conducted through quality assurance (QA) and quality control (QC) [20,21]. Both seawater and melting ice samples were filtered through polycarbonate filters with a pore size of 0.45 μm before the dissolved trace metal concentration was measured. The detection methods for trace metals copper (Cu), lead (Pb), cadmium (Cd), zinc (Zn), and chromium (Cr) are using all atomic absorption spectrophotometry (TAS-990 Super AFG). The concentrations of Cu, Pb, Cd and Cr were measured by atomic absorption spectrometry (AAS), and the concentration of zinc (Zn) was analyzed using graphite furnace atomic absorption spectrometry (GFAAS). The total and dissolved mercury (Hg) concentrations in seawater and ice samples were measured using atomic fluorescence spectrophotometer (AFS-9130). The relative standard deviations for precision were 3.8%, 4.0%, 3.5%, 2.6%, 3.1% and 2.2% for Cu, Pb, Cd, Zn, Cr and Hg, respectively. Spike recoveries ranged from 87.6% to 110.2%.

The partition coefficient (*K*_d_) of a particular trace metal between the particulate and dissolved fractions is defined as the ratio of the trace metal fraction in the particles ([Me_p_], in μg/g) to that in the dissolved concentration ([Me_d_], in μg/L), as given in Equation (1). For the convenience of comparison, the logarithmic value is usually used.

(1) $$K_{d}=\frac{\left[{\mathrm{Me}}_{p}\right]}{\left[{\mathrm{Me}}_{d}\right]}$$

## 3. Results

### 3.1. Spatial Distribution of Trace Metals

**Figure 2:**
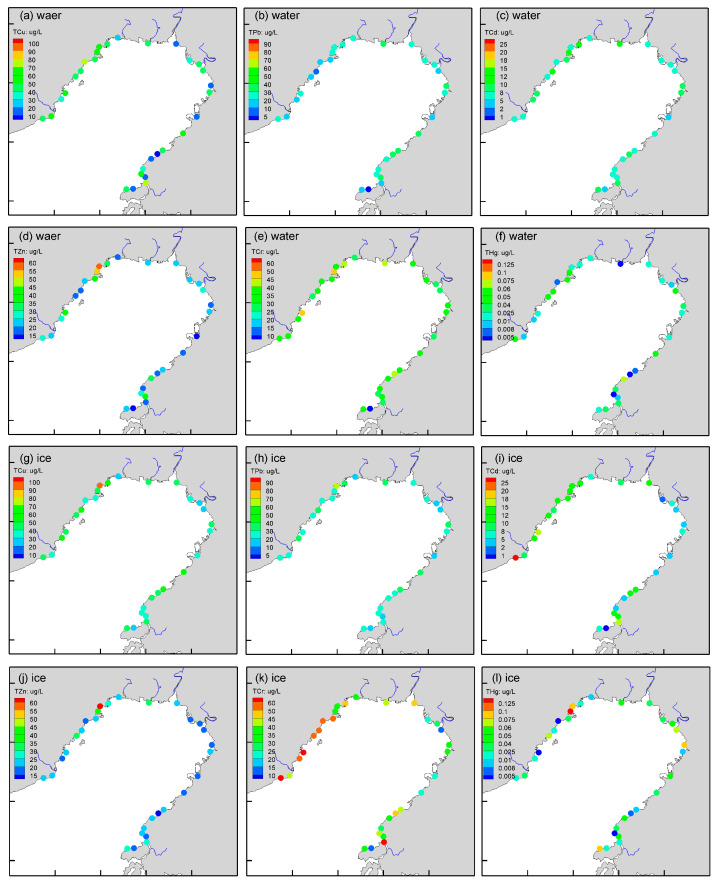
Spatial distribution of trace metals in water and ice around Liaodong Bay. (**a**) TCu in seawater; (**b**) TPb in seawater; (**c**) TCd in seawater; (**d**) TZn in seawater; (**e**) TCr in seawater; (**f**) THg in seawater; (**g**) TCu in ice; (**h**) TPb in ice; (**i**) TCd in ice; (**j**) TZn in ice; (**k**) TCr in ice; (**l**) THg in ice.

The concentrations of total copper (TCu), lead (TPb), cadmium (TCd), zinc (TZn), chromium (TCr) and mercury (THg) in the water range between 8.78–76.70 (avg. 40.65 ± 18.63), 2.92–33.21 (avg. 21.85 ± 8.26), 3.34–13.06 (avg. 7.78 ± 2.07), 11.57–58.76 (avg. 25.99 ± 10.63), 9.30–48.64 (avg. 34.19 ± 7.68), and 0.002–0.064 (avg. 0.025 ± 0.019) μg/L, respectively (Figure 2). The average total metal concentration is in the order TCu > TCr > TZn > TPb > TCd > THg. Due to the great differences in emission intensity and water dilution capacity, the concentration of trace metals varies significantly across regions. The coefficients of variation (CV) of TCu, TPb, TCd, TZn, TCr and THg in seawater are 45.8%, 37.8%, 26.5%, 40.9%, 22.5% and 69.9%, respectively.

Concentrations of TCu, TPb, TCd, TZn, TCr and THg in the ice range between 23.51–98.49 (avg. 41.81 ± 14.72), 14.97–62.17 (avg. 26.92 ± 9.22), 0.66–25.33 (avg. 9.21 ± 5.28), 14.83–64.68 (avg. 23.80 ± 9.77), 10.66–72.76 (avg. 40.28 ± 15.28), and 0.002–0.130 (0.038 ± 0.031) μg/L, respectively. The average total metal concentration is in the order Cu > Cr > Pb > Zn > Cd > Hg. The coefficients of variation (CV) of TCu, TPb, TCd, TZn, TCr and THg in sea ice are 35.2%, 34.3%, 57.4%, 41.1%, 37.9% and 82.7%, respectively. Higher inter-sample variability of Cd, Cr, and Hg are found in ice phases, and variability of Cu in ice samples is significantly lower than that in seawater.

There are many cities along Liaodong Bay, and the Daliao River, Liaohe River, Daling River, etc., that flow into this area, gathering a large number of terrigenous materials from the drainage basin. In spring, summer and autumn, the concentration of trace metals in the northern, top area of Liaodong Bay is generally higher than that in the eastern and western coastal seas of Liaodong Bay. Nevertheless, this spatial feature is not obvious in winter. The type of sea function is a more important factor in determining the levels of trace metals. The concentrations of heavy metals in the industrial sea area are significantly higher than that in other functional areas [22].

The mean ratios of total trace metals between sea ice and seawater are 1.41 (Cu), 1.51 (Pb), 1.21 (Cd), 0.97 (Zn), 1.20 (Cr) and 2.48 (Hg), respectively. It seems that most trace metals are enriched in sea ice. The *t* test is used to further statistically analyze the metal levels in two phases. It is found that ice holds significantly higher levels (*p* < 0.05, *n* = 30) of TCr compared to water. There are no significant differences in concentrations of TCu, TPb, TCd, TZn and THg between the water and ice samples (*p* > 0.05, *n* = 30).

The redistribution effect of sea ice on total concentrations of trace metals does not seem to be significant. There is neither significant enrichment of heavy metals such as polar sea ice nor obvious extrusion effect like that of freshwater lakes [14,23]. The migration of trace metals between ice and water is consistent with Lannuzel’s report on Antarctic pack ice [24]. In the presence of complicated chemical speciation, the partitioning of various trace metals between seawater and ice is complex. Sediments incorporated into newly frozen ice strongly influence the transport and distribution of heavy metals. The concentrations of all types of particulate metals are higher in ice than in seawater, while the dissolved phase ratios decline. It should be said that the redistribution of heavy metals by sea ice is uncertain, and that the migration results of different heavy metals may vary greatly. Even for the Antarctic ice, the trace metals such as Cu, Cd, Zn and Cr do not appear to be as particularly enriched in the sea as lithogenic element iron (Fe) [24]. More importantly, the effects on different chemical speciations of a trace metal may also be diametrically opposite. Natural processes (e.g., wind drift, tidal currents, and sea-level rise) and environmental factors (e.g., suspended particles, salinity, and acidity) can also affect the metal redistribution.

Another overlooked factor is that the concentration of trace metal in Liaodong Bay is much higher than that in polar seas. Considering the concentration effect, the issue of whether the migration law of trace metals between ice and seawater is applicable to Liaodong Bay needs further study.

Other than trace metal, some other seawater properties are measured, such as COD (chemical oxygen demand using alkaline KMnO_4_), salinity (sal.), and suspended particulate material (SPM). COD of seawater ranges from 0.09 to 1.98 mg/L, with an average of 0.58 mg/L, while it varies from 0.14 to 2.83 mg/L in the ice, on an average of 0.83 mg/L. The salinity (Sal.) of coastal waters in the survey averaged 31.3 and below 30 during the ice-free period. Suspended particulate material (SPM) concentrations in Liaodong Bay are high and range between 20.1–146.2 mg/L. Concentrations of SPM in seawater ranges from 0.01 to 0.16 mg/L, on an average of 0.06 mg/L (*n* = 30), values which are in good agreement with those previously found in the nearshore waters of Liaodong Bay. Concentrations of SPM in the sea ice are about 4 times higher than those in seawater, ranging from 1.0 to 1544 mg/L, with an average of 156.8 mg/L (*n* = 30).

### 3.2. Partitioning of Trace Metals in Coastal Water and Sea Ice

**Figure 3:**
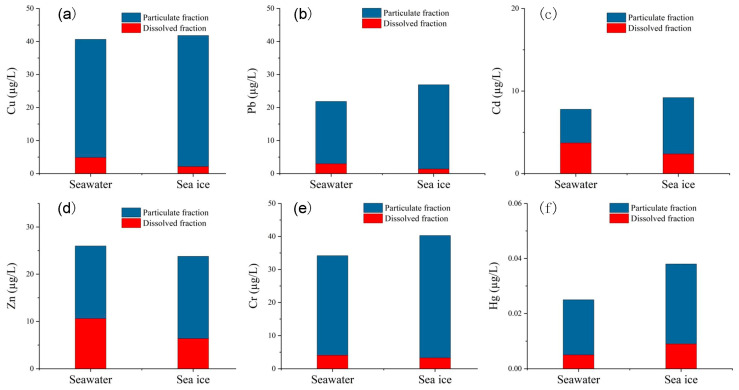
The average concentrations of particulate and dissolved heavy metals in seawater and sea ice over 30 coastal sites in Liaodong Bay. (**a**) Cu; (**b**) Pb; (**c**) Cd; (**d**) Zn; (**e**) Cr; (**f**) Hg.

As shown in Figure 3, concentrations of particulate copper (PCu), lead (PPb), cadmium (PCd), zinc (PZn), chromium (PCr) and mercury (PHg) in the seawater range from 4.88–71.91 (avg. 35.83 ± 18.07), 1.78–30.37 (avg. 18.88 ± 8.04), 0.21–8.16 (avg. 4.08 ± 1.93), 5.23–35.91 (avg. 15.40 ± 7.22), 4.87–44.74 (avg. 30.12 ± 7.85), and 0.001–0.058 (avg. 0.020 ± 0.018) μg/L, respectively. Concentrations of dissolved copper (DCu), lead (DPb), cadmium (DCd), zinc (DZn), chromium (DCr) and mercury (DHg) in the seawater range from 1.36–13.6 (avg. 4.82 ± 2.67), 0.51–15.10 (avg. 2.97 ± 2.65), 1.81–5.03 (avg. 3.71 ± 0.91), 3.82–24.84 (avg. 10.59 ± 5.52), 0.77–10.07 (avg. 4.07 ± 1.67), and 0.001–0.013 (avg. 0.005 ± 0.004) μg/L, respectively. The dissolved-to-total metal ratio of Cu, Pb, Cd, Zn, Cr and Hg in seawater are 14.7%, 15.2%, 50.1%, 40.7%, 13.1% and 23.5%, respectively. With the exception of Cd, heavy metals in seawater are primarily found in the particulate phase. The concentration of dissolved trace metals in seawater decreased significantly compared with that before freezing (Guo et al., 2022). As the dry season comes, the riverine input drops sharply, and the trace metals entering Liaodong Bay also decrease greatly. The wind speed is usually high in winter, and the sea water is mixed evenly. Strong sea condition contributes to the rapid dispersion of pollutants in the nearshore water to the offshore sea, decreasing the spatial variability of trace metals.

Concentrations of PCu, PPb, PCd, PZn, PCr and PHg in the sea ice range from 22.37–96.84 (avg. 39.70 ± 14.73), 13.74–59.75 (avg. 25.55 ± 9.31), 0.36–21.59 (avg. 6.84 ± 4.96), 8.98–51.71 (avg. 17.44 ± 8.57), 7.35–65.67 (avg. 37.02 ± 14.51), and 0.001–0.079 (avg. 0.029 ± 0.024) μg/L, respectively. Concentrations of DCu, DPb, DCd, DZn, DCr and DHg in the sea ice range from 0.52–4.99 (avg. 2.11 ± 1.24), 0.42–4.81 (avg. 1.37 ± 1.15), 0.28–4.36 (avg. 2.37 ± 1.10), 2.52–12.97 (avg. 6.36 ± 2.39), 1.79–11.76 (avg. 3.26 ± 2.34), and 0.001–0.051 (avg. 0.009 ± 0.012) μg/L, respectively. The dissolved-to-total metal ratios of Cu, Pb, Cd, Zn, Cr and Hg in sea ice are 5.5%, 5.7%, 32.1%, 27.7%, 9.0% and 24.4%, respectively. It is found that all studied trace metals of dissolved phase are found almost exclusively from coastal ice in Liaodong Bay. The ratios of particulate heavy metals between sea ice and seawater are 1.73 (Cu), 1.80 (Pb), 1.97 (Cd), 1.25 (Zn), 1.27 (Cr) and 2.65 (Hg), respectively. It can be seen that all heavy metal particulate states are concentrated in ice.

The ratios of dissolved trace metals between sea ice and seawater were 0.52 (Cu), 0.54 (Pb), 0.65 (Cd), 0.67 (Zn), 0.95 (Cr) and 3.12 (Hg), respectively. It can be seen that all metals of dissolved states, except Hg, are expelled during the process of freezing. Concentrations of DCu, DPb, DCd and DZn are significantly lower in sea ice compared to the ambient water. It has been previously reported that the concentration of DCd in ice is lower than that in seawater [25]. In contrast to other elements, higher concentrations of DHg are observed in sea ice, which may be due to its volatile property in the liquid phase.

## 4. Discussion

### 4.1. Factors Affecting Trace Metal Distribution

The source and distribution of heavy metals in the coastal area are mainly attributed to human activities (emissions of industrial production, residues of agricultural activities, domestic sewage, atmospheric deposition) and natural factors (input of natural weathering products from the basin, hydrodynamic conditions). By means of a correlation analysis, we can speculate whether the sources of heavy metals are consistent or whether the geochemical properties of elements are similar.

The relationship between the six metals and the physicochemical properties of seawater is investigated using simple linear regression (Table 1). A coefficient of over 0.2 is usually regarded as statistically significant, with *p* < 0.05 level. Aside from Hg, all elements have positive significant correlation with each other, which indicates that their sources and environmental behaviors are the same or similar, and they are affected by human activities to a similar extent.

Enhanced correlation between trace metals in sea ice exhibits their similar migration and redistribution during ice formation (Table 2), and all the coefficients are positive. There is a significant correlation between Pb, Zn and Ni, which may be caused by the great difference in the geochemical behavior of the three metals. TCu concentrations are negatively correlated with TPb in the water phase but positively correlated in ice. This homoplasy is generated by the combination of the strongly organically bound nature of Cu and the surface-reactive property of Pb [26]. TCr is significantly correlated with TPb, TCd, and TZn and the highest coefficient is with TCd (0.77). THg is negatively correlated with all other metals due to its unique biogeochemical process and phase state in coastal environments. Ice enrichment factors of chemical oxygen demand (COD) are greater than 1 at 73% of the sampling sites, and the mean enrichment factor is 2.59. The COD survey results are consistent with the previous PAHs results [27], indicating that organic matters tend to accumulate in ice (selective incorporation of dissolved organic matter). Most metals, except for Hg, are negatively correlated with COD, but the correlation is low and non-significant. The reason behind this is not difficult to understand, as most trace metals mainly exist in suspended sediments in coastal waters. On the contrary, the filter-passing dissolved faction is increasing with COD due to the form of a strong organic complex.

Sediment particles are instrumental in controlling the transport processes between the water column and bed sediments. On one hand, the concentration of particulate trace metals increases with the increase in the concentration of suspended sediments in water. On the other hand, the increase in the concentration of suspended sediments will produce a “particle concentration effect”, reducing the adsorption capacity of particles to heavy metals [26]. The two opposite processes lead to extremely complex results, and there is no significant correlation between the concentration of SPM and trace metals (except for TCu) in water.

SPM concentrations in ice range between 1.1–1544 mg/L, approximate 4.3 times the variation in water. Sediment-laden sea ice is a ubiquitous phenomenon in the frozen seas, and it covers more than a half faction of the total ice coverage, with a maximum of 80–90% in Liaodong Bay. The sea ice in Liaodong Bay is first formed near the northern coastline. Under the action of tide, current and wind, sea ice frequently scrapes the seabed, and a large amount of sediment enters the ice body. However, there is no corresponding increase in the levels of trace metals in sea ice, because the nearshore sediment size is relatively large with low adsorption of heavy metals [28].

The salinity (Sal.) of coastal waters in the survey averaged 31.3 and was below 30 during the ice-free period. With increasing salinity in water, most trace metals (except for Hg) exhibited an evident rise, especially for Pb, Cd, and Cr. A considerable number of dissolved trace metals, like salts, are redistributed from ice to the surrounding water during the freezing process. Moreover, the increase in salinity will produce a “salinity effect” and promote the desorption of trace metal ions from suspended particles [29].

### 4.2. Trace-Metal Partitioning, K_d_

**Figure 4:**
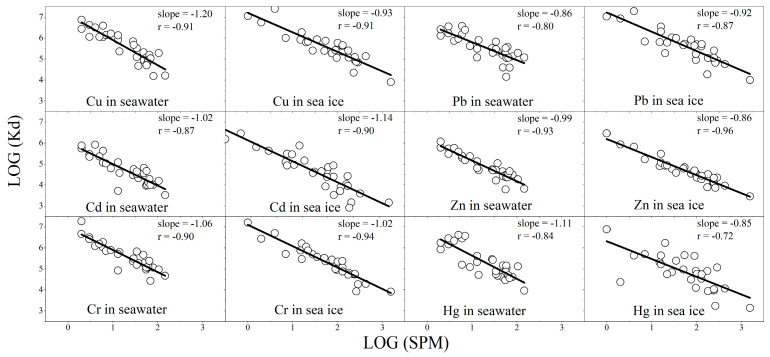
Partition coefficients for trace metals for all 30 coastal sites as a function of SPM.

Values of *K*_d_ in both water and ice phases are plotted against SPM concentrations on a logarithmic scale in Figure 4. The average values of *K*_d_ in the water phase increase in the order of Cd < Zn < Hg < Cu < Pb < Cr, consistent with previous results from Galveston Bay [26], while the average values of *K*d in the ice phase are in the order of Cd < Zn < Hg < Cr <Cu < Pb.

For *K*_d_ of Cu and Cd, there is no evident difference between water and ice. The flux of atmospheric deposition Pb is greater than the river discharge in Liaodong Bay [30]. In winter, because the sea surface is icebound, Pb from atmospheric sedimentation is mainly stored in ice as a particle state. This is why the value *K*_d_ of Pb rises in ice. The *K*_d_ values of Zn, Cr, and Hg decline in the ice phase, but for different reasons. Dissolved Hg in ice is relatively more difficult to volatilize into the atmosphere than that in water, resulting in its higher dissolved content in ice. Due to the rubbing between sea ice and the seabed, a large amount of sediment with large size on the seabed is mixed, which increases the concentration of SPM without increasing the corresponding particulate Cr. The enrichment of organic matter in ice increases the chance of Zn bonding with organic matter to become dissolved state.

An evident negative relationship between the particulate/solution partition coefficient of all trace metals and suspended particulate matter can be seen in Figure 4. High particle concentrations lead to the universal decrease in the scavenging of trace metals, called the “particle concentration effect” (PCE) [31], which is attributed to heterogeneity effects of sediment size and particle composition. There is relatively less dependence of *K*_d_ on SPM concentrations for Pb, Cd, Zn, and Hg, but there are large changes for Cu and Cr. It appears that PCE for each metal is changeable in other estuaries and coastal regions. Wen et al. (1999) found that it is noticeable only for Cu, and that the *K*_d_ values of Pb, Cd, and Zn were nearly independent of SPM concentrations [32]. PCE varies greatly for different elements, as the value of Pb and Cd increased and that of Cu, Zn, Cr, and Hg decreased.

## 5. Conclusions

We reported the concentrations of trace metals (Cu, Pb, Cd, Zn, Cr and Hg) in both water and ice to understand the sea ice environment of Liaodong Bay. Compared with other seasons, the pollution level of trace metals in Liaodong Bay is lower in winter. Decreasing terrestrial input and strong mixed effect of water contribute to a decline in spatial variability.

This study shows that there is a varied enrichment for different trace metals during the freezing of seawater, which is probably caused by their physico-chemical properties and environmental factors. Except for Cr, the rest of the five metals do not appear to be significantly enriched in sea ice as compared to seawater. In general, the dissolved levels of all metals are elevated as a result of desalination, with the exception of Hg, and the particulate levels are elevated due to sediment entrainment. However, the partitioning of these trace metals does not show strong bias towards the particulate phase in ice, because incorporated incorporation bed sediments with large size absorb relatively few trace metals.

It should be pointed out that the sampling sites in this survey are all located nearshore. To understand the impact of sea ice on the environmental behavior of trace metals, the investigation scope should be extended to offshore areas. Further attention should also be paid to the relationship between the trace metals and other substances, such as nutritional components and persistent organic pollutants.

## Figures and Tables

**Table 1:** Person correlation matrix for total heavy metal concentrations and the physicochemical parameters in seawater.

	Cu	Pb	Cd	Zn	Cr	Hg
Cu	1					
Pb	−0.95	1				
Cd	0.48 **	0.22	1			
Zn	0.25	0.22	0.44 *	1		
Cr	0.23	0.46 **	0.77 **	0.39 *	1	
Hg	−0.02	−0.15	−0.19	0.16	−0.22	1
COD	−0.04	−0.24	−0.26	−0.27	−0.35	0.15
Sal.	0.15	0.58 **	0.57 **	0.246	0.79 **	−0.12
SPM	−0.44 *	0.082	0.045	0.12	0.077	0.004

* Correlation is significant at the 0.05 level (2-tailed). ** Correlation is significant at the 0.01 level (2-tailed).

**Table 2:** Person correlation matrix for total heavy metal concentrations and the physicochemical parameters in sea ice.

	Cu	Pb	Cd	Zn	Cr	Hg
Cu	1					
Pb	0.99 **	1				
Cd	0.29	0.29	1			
Zn	0.72 **	0.72 **	0.17	1		
Cr	0.33	0.34	0.88 **	0.13	1	
Hg	0.29	0.28	0.12	0.59 **	0.10	1
COD	0.43 *	0.44 *	−0.28	0.51 **	−0.03	0.50 **
Sal.	0.28	0.28	0.92 **	0.07	0.91 **	−0.05
SPM	0.23	0.24	−0.12	0.38 *	−0.10	0.15

* Correlation is significant at the 0.05 level (2-tailed). ** Correlation is significant at the 0.01 level (2-tailed).

## Data Availability

The data presented in this study are available on request from the corresponding author.
